# In‐depth immune profiling of peripheral blood mononuclear cells in patients with pancreatic ductal adenocarcinoma reveals discriminative immune subpopulations

**DOI:** 10.1111/cas.16147

**Published:** 2024-04-30

**Authors:** Ernesto Rodriguez, Eline S. Zwart, Alsya A. Affandi, Jan Verhoeff, Mike de Kok, Lenka N. C. Boyd, Laura L. Meijer, Tessa Y. S. Le Large, Katarzyna Olesek, Elisa Giovannetti, Juan J. García‐Vallejo, Reina E. Mebius, Yvette van Kooyk, Geert Kazemier

**Affiliations:** ^1^ Department of Molecular Cell Biology and Immunology Amsterdam UMC, VU University Amsterdam Amsterdam The Netherlands; ^2^ Amsterdam Institute for Infection and Immunity, Cancer Immunology Amsterdam The Netherlands; ^3^ Cancer Center Amsterdam, Cancer Biology and Immunology Amsterdam The Netherlands; ^4^ Department of Surgery Amsterdam UMC, VU University Amsterdam Amsterdam The Netherlands; ^5^ Department of Medical Oncology Amsterdam UMC, VU University Amsterdam Amsterdam The Netherlands; ^6^ Cancer Pharmacology Lab, AIRC Start‐Up Unit Fondazione Pisana per la Scienza Pisa Italy

**Keywords:** biomarker, immunophenotyping, mass cytometry, pancreatic cancer, PBMC

## Abstract

Pancreatic ductal adenocarcinoma (PDAC) has a dismal prognosis with a 5‐year survival of less than 10%. More knowledge of the immune response developed in patients with PDAC is pivotal to develop better combination immune therapies to improve clinical outcome. In this study, we used mass cytometry time‐of‐flight to undertake an in‐depth characterization of PBMCs from patients with PDAC and examine the differences with healthy controls and patients with benign diseases of the biliary system or pancreas. Peripheral blood mononuclear cells from patients with PDAC or benign disease are characterized by the increase of pro‐inflammatory cells, as CD86^+^ classical monocytes and memory T cells expressing CCR6^+^ and CXCR3^+^, associated with T helper 1 (Th1) and Th17 immune responses, respectively. However, PBMCs from patients with PDAC present also an increase of CD39^+^ regulatory T cells and CCR4^+^CCR6^−^CXCR3^−^ memory T cells, suggesting Th2 and regulatory responses. Concluding, our results show PDAC develops a multifaceted immunity, where a proinflammatory component is accompanied by regulatory responses, which could inhibit potential antitumor mechanisms.

## Graphical abstract

This study used mass cytometry to undertake an extensive characterization of the circulating immune cells in patients with pancreatic ductal adenocarcinoma (PDAC). We show that PBMCs from patients with PDAC are characterized by the increase of proinflammatory cells, as CD86^+^ classical monocytes and memory T cells expressing CCR6^+^ and CXCR3^+^, associated with T helper 1 (Th1) and Th17 immune responses, respectively. However, PBMCs from patients with PDAC also show an increase of CD39^+^ regulatory T cells and CCR4^+^CCR6^−^CXCR3^−^ memory T cells, suggesting Th2 and regulatory responses.

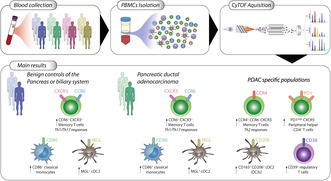

**Abbreviations**
BMI: body mass index
CA19‐9: carbohydrate antigen 19‐9
cDC2: conventional dendritic cell type 2
CyTOF: cytometry time‐of‐flight
DC: dendritic cell
IFNγ: γ‐interferon
IL: interleukin
MDSC: myeloid‐derived suppressor cell
NK: natural killer
PD‐1: programmed cell death‐1
PDAC: pancreatic ductal adenocarcinoma
pDC: plasmacytoid dendritic cell
PD‐L1: PD‐1 ligand
Th: T helper
TME: tumor microenvironment
TNFα: tumor necrosis factor α
Tph: T peripheral helper
T_RM_
: resident memory T

## 1 INTRODUCTION

Pancreatic ductal adenocarcinoma is an aggressive malignancy with a 5‐year survival of less than 10%.^1^ Most patients are considered irresectable at the time of diagnosis, either due to distant metastasis or locally advanced disease. These patients are solely dependent on systemic therapy, consisting mostly of chemotherapy, with limited effect on survival and potential severe side‐effects.^2^, ^3^, ^4^ One reason for this limited effect of chemotherapy is the immune suppressive microenvironment with dense fibrotic stroma protecting the tumor cells.^5^ However, chemically removing this dense fibrotic stroma has led to controversial results, suggesting that stroma also has tumor‐restraining properties.^6^ Indeed, single cell analysis of murine PDAC stromal cells identified distinct, and potentially functional, subtypes of cancer‐associated fibroblasts.^7^ Focus has thus shifted toward overcoming the tolerogenic TME.

Immune cells present in the PDAC TME consists mostly of regulatory T cells, M2 macrophages and MDSCs.^8^, ^9^ The ratio between the immune suppressive cells and the proinflammatory cells, but also the distance between these and tumor cells, are correlated with the survival of patients.^9^, ^10^, ^11^, ^12^, ^13^ Several clinical trials have been undertaken to enhance immunological mediators and suppress tumor cells. Patients with PDAC are particularly refractory to novel immunotherapies with immune checkpoint inhibitors such as PD‐1, CTLA‐4, and PD‐L1.^14^, ^15^, ^16^ The hypothesis is that high tumor burden prevents PDAC from being cured by a monotherapy such as PD‐1/PD‐L1 blockade alone. This does not hold true for mismatch repair deficient PDAC, which only constitutes 0.8%–1.2% of all tumors.^17^, ^18^, ^19^ However, combination therapies have shown promise, as these therapies can have a synergistic effect, leading to, for example, more tumor antigens in case of chemo(radio)therapy or more T cells close to the tumor cells in case of GVAX vaccines.^20^

Pancreatic ductal adenocarcinoma is also able to affect the immune system at a systemic level and this may reflect changes observed locally.^21^ It has recently been described that the expression levels of TIGIT, but not of PD‐1, in CD8^+^ T cells present in tissue are correlated with the ones found in PBMCs.^21^ Moreover, the levels of regulatory T cells in blood of patients with PDAC correlate with response to the chemotherapeutic drug gemcitabine, where levels significantly decreased in patients with stable disease or partial response to therapy.^22^, ^23^ Therefore, a better characterization of circulating immune cells present in PDAC can serve as a tool for patient stratification and monitoring but potentially also for the development of better combination immune therapies.

Moreover, characterization of PBMCs in PDAC could not only be important to discover novel treatment options, but also serve as a diagnostic biomarker. Most patients with PDAC are diagnosed in late stages of the disease, eliminating curative options, which shows the need for the development of early detection tools. To date, CA19‐9 is currently the only biomarker used in the clinic for PDAC, but due to its low specificity and sensitivity, it is mostly used to monitor already diagnosed patients.^24^, ^25^ Many studies have shown that a combination of biomarkers or a complete array appears to provide the highest specificity and sensitivity.^26^, ^27^, ^28^

Previous reports have shown the potential power of immune signatures as biomarkers to contribute to diagnose cancer.^29^ Therefore, we aimed to perform an in‐depth characterization of circulating immune cells in blood from patients with PDAC and examine the differences with healthy controls and patients with benign diseases of the biliary system or pancreas. To achieve this, we undertook CyTOF, using two extensive panels, each containing 38 markers, and focused on markers associated with lymphoid or myeloid populations. With the use of dimensional reduction and unbiased clustering algorithms, we were able to generate the most comprehensive overview of circulating immune cells in PDAC to date, identifying 60 immune populations.

## 2 MATERIALS AND METHODS

### 2.1 Patient samples

The study design and protocol were approved by the local Medical Ethics Board of the Amsterdam UMC, VU University Amsterdam in accordance with the ethical guidelines of the Declaration of Helsinki. Written informed consent was obtained from all participants before study participation. Patients with PDAC and benign diseases including pancreatitis, cholecystolithiasis, intraductal papillary mucinous neoplasm, cholangitis, choledocholithiasis, lipoma of the pancreas, benign stenosis of the common bile duct, and pancreatic cysts were included between July 2017 and January 2019. All patients with PDAC were histopathologically confirmed by an independent pathologist. Blood from the studied patients was obtained prior to start of treatment. For the isolation of PBMCs, whole blood was drawn in BD Vacutainers (Becton Dickinson) containing K2 EDTA, while BD Vacutainers with no anticoagulant or preservative were used to obtain the serum. Healthy controls were included in December 2016. Patients were excluded if they were diagnosed with another malignancy less than 5 years ago (excluding basal cell carcinoma), had immunological diseases, or received immunosuppressive drugs. Clinicopathologic characteristics were collected in a prospectively maintained database. Four groups were formed, consisting of patients with resectable PDAC, patients with irresectable PDAC (composed of patients with locally advanced PDAC or metastatic PDAC), patients with benign diseases of the pancreas or biliary system (henceforth referred to as “benign controls”), and healthy controls.

### 2.2 Isolation of PBMCs

For the isolation of PBMCs, blood was first spun down for 20 min at 120 *g* to separate the platelet‐enriched plasma and PBMCs, which were collected from buffy coats by using a Lymphoprep gradient (Stemcell Technologies). Within each CyTOF staining and acquisition, one reference sample was included to be able to normalize for staining intensity differences between different batches. This sample was obtained from the buffy coat of one healthy control which was aliquoted after processing. Cells were cryopreserved in liquid nitrogen until their use in this study. The PBMCs were resuscitated by rapid thawing, addition of 15 mL prewarmed RPMI medium complemented with 20% FCS, and subsequently washed with Maxpar PBS (Fluidigm) before staining for the CyTOF protocol or PBS containing 2% BSA and 0.05% sodium azide for the flow cytometry protocol. Serum was obtained by letting the whole blood clot at room temperature and centrifugation at 2000 *g* for 10 min. All serum samples were frozen at −80°C until use.

### 2.3 Clustering and dimensional reduction

For clustering and immune population discovery, two rounds of FlowSOM clustering were performed using the function “cluster” as applied in the CATALYST package, defining 400 clusters and 100 metaclusters (Figure S1).^30^ The first clustering allowed us to identify markers that define cell *types* and the ones that define cell *states*. *Type* markers were used in a second FlowSOM clustering to define cell populations, which were later confirmed by manual gating. Dimensional reduction was performed with the function “runDR”. After the identification of immune subpopulations, we analyzed the differential expression of *state* markers by using the package “diffcyt”.

### 2.4 Statistical analysis

Clinical data were analyzed in SPSS (IBM SPSS Statistics version 26). For nonskewed continuous data, the one‐way ANOVA test was used and reported with the mean and SD. For skewed continuous data, the Kruskal–Wallis test was used and reported with the median and interquartile range. Pearson's χ^2^‐test was used for ordinal and nominal data. For the analysis of the blood markers (leukocytes, hemoglobin, bilirubin, and CA19‐9), the compositional data were centered log ratio transformed based on percentages, followed by an ANOVA test to detect differences between the patient groups. A *p* value ≤0.05 was considered significant.

For the differential discovery of immune populations, a linear model was used. Frequencies of each cell type in total CD45^+^ cells and in parental cell type were used as variables. Furthermore, the most relevant differentially expressed markers were quantified by manual gating. Size effect was estimated by calculating the difference between population means and dividing by the SD of controls.

To study the associations between the different immune populations, Spearman correlations between the frequencies of the different population in total CD45^+^ cells were calculated separately for benign disease and resectable and irresectable PDAC. Results were displayed as networks using the packages “igraph” and “ggraph”. Only correlations with an absolute Spearman coefficient ≥0.5 and a *p* value ≤0.01 were displayed.

## 3 RESULTS

### 3.1 Patient characteristics

Clinical data of the patients and healthy controls analyzed in this paper are available in Table S1. Different compositions of patients were included per analysis, due to the predefined cut‐off of 20 000 CD45^+^ cells. There were no significant differences in the age or sex of patients with resectable or irresectable PDAC, benign controls, and healthy controls. In the CyTOF panels, 10 patients with irresectable PDAC underwent biliary drainage prior to blood withdrawal, compared to 9 patients with resectable PDAC and 3 patients with benign diseases. In addition, 7 patients with irresectable PDAC underwent an endoscopic ultrasound with fine needle aspiration prior to blood withdrawal, compared to 4 patients with resectable PDAC and 3 patients with benign diseases. Three patients with irresectable PDAC had a tumor located in the tail compared to 2 patients with resectable PDAC. All other tumors were in the head of the pancreas. Patients with benign diseases underwent significantly less biliary drainage, and had a significantly higher BMI and hemoglobin level and a lower CA19.9 and bilirubin level. Finally, patients with resectable PDAC had a significantly higher leucocyte count (Table S1).

### 3.2 Panel design and characterization

**FIGURE 1:**
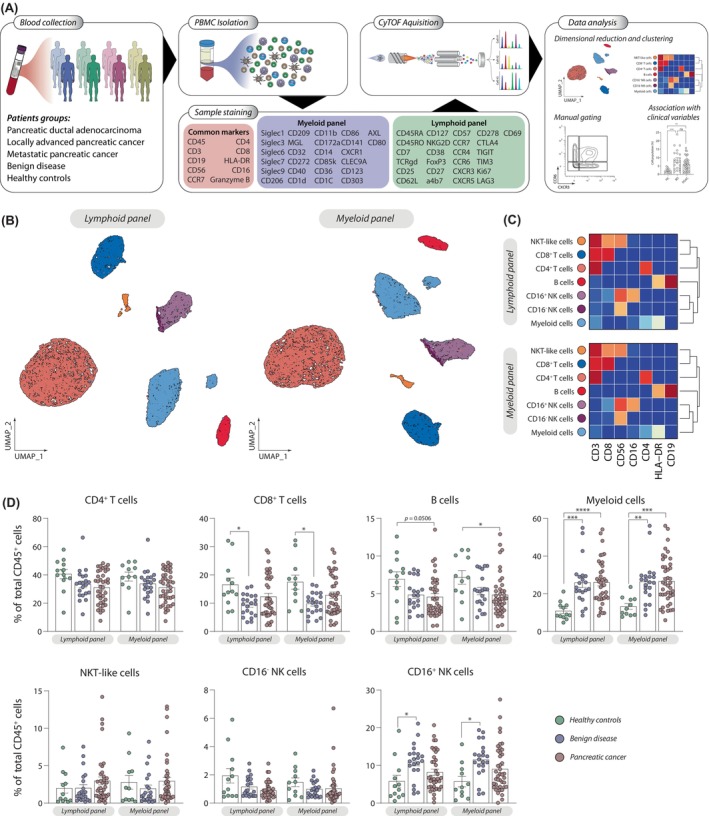
Evaluation of the cytometry time‐of‐flight (CyTOF) panels used in this report. (A) Workflow for the deep immunophenotyping of PBMCs in pancreatic ductal adenocarcinoma (PDAC). (B) UMAP plots and (C) heatmaps using common markers in the myeloid (M) and lymphoid (L) panels and colored based in manual gated immune populations. (D) Quantification of the gated immune populations in healthy controls (lymphoid, *n* = 12; myeloid, *n* = 11), benign disease (lymphoid, *n* = 21; myeloid, *n* = 21), and PDAC (lymphoid, *n* = 39; myeloid, *n* = 42). Pairwise comparisons: *p ≤ 0.05, **p ≤ 0.01, ***p ≤ 0.001, ****p ≤ 0.0001, Kruskal–Wallis test. NK, natural killer.

For the generation of a comprehensive overview of the PBMCs, we designed two different CyTOF panels (Figure 1A, Tables S2 and S3) that focus on the characterization and quantification of a broad variety of myeloid and lymphoid cell subsets (Figure 1A). We made use of the common markers (CD3, CD19, HLA‐DR, CD56, and CD16) to undertake an initial characterization and comparison of the two panels, by performing manual gating of general immune populations (Figures 1B–D and S2A,B). Our data showed that the quantification performed on the myeloid and lymphoid panel resulted in similar cell frequencies of the different populations analyzed, observing a strong correlation between both panels (Figures 1C,D and S2C). This preliminary analysis reveals a strong increase of circulating myeloid cells (CD45^+^CD3^−^CD19^−^CD56^−^HLA‐DR^+^ cells) in both PDAC patients and those with benign disease, compared to healthy individuals (Figure 1D). Moreover, we observed a decrease of B cells in patients with PDAC, while patients with benign disease, show a decrease of CD8^+^ T cells and an increase of CD16^+^ NK cells (Figure 1D).

### 3.3 Characterization of myeloid cells reveals a specific increase of CD206 ^+^ cDC2 in PDAC

**FIGURE 2:**
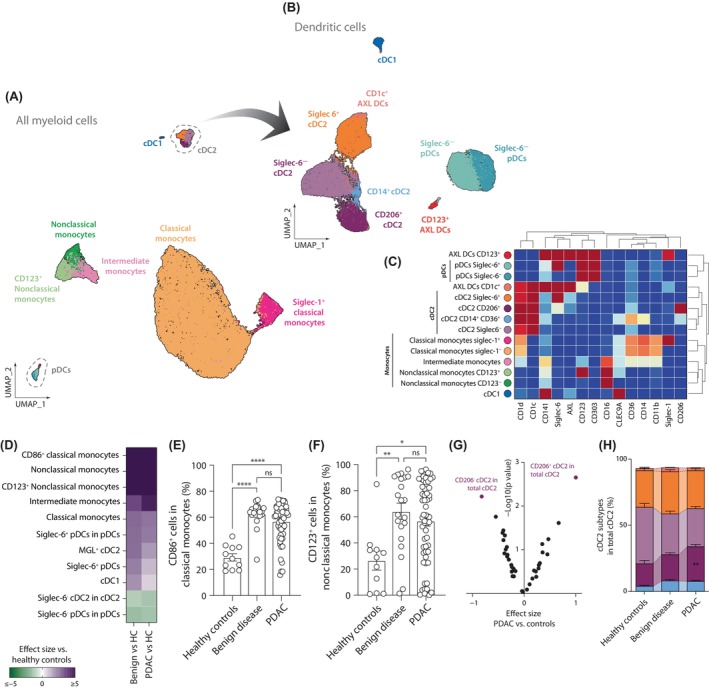
Characterization of circulating myeloid cells. UMAP plots showing (A) all the myeloid cells or (B) only dendritic cells (DC) identified. (C) Heatmap showing the expression of the different “type” markers in all the myeloid subpopulations. (D) Heatmap summarizing the effect size of significantly abundant populations in patients with pancreatic ductal adenocarcinoma (PDAC) or benign disease against healthy controls. (E, F) Percentages of (E) CD86^+^ and (F) CD123^+^ in classical and nonclassical monocytes, respectively. (G) Volcano plot of the *p* value and the effect size of the differential cell abundance in PDAC compared to controls (healthy and benign disease). (H) Quantification of the different conventional DC2 (cDC2) subtypes in healthy controls, benign disease, and PDAC. Pairwise comparisons: *p ≤ 0.05, **p ≤ 0.01, ***p ≤ 0.001, ****p ≤ 0.0001, Kruskal–Wallis test. ns, not significant; pDC, plasmacytiod DC.

To increase the chances of identifying rare populations, we opted for performing individual analysis in the general populations described before, starting with myeloid cells using only the myeloid panel. For this, we gated myeloid cells (defined as CD3^−^CD19^−^CD56^−^HLA‐DR^+^) and performed FlowSOM clustering and dimensional reduction for the identification of different immune populations, which were confirmed and quantified by manual gating (Figures S1 and S2A,B). This approach led to the identification of 14 different myeloid subtypes, including two different subtypes of AXL^+^ DCs that were present in low frequencies in peripheral blood (Figure 2A–C).

We observed a great diversity in cDC2, identifying four different populations that resembled recently reported cDC2 subpopulations.^31^, ^32^ For example, the *Siglec‐6* ^+^ *cDC2* identified in our analysis were associated with CD5^+^ cDC2, which have been shown to express the gene *SIGLEC6*.^32^, ^33^ We could also find cells linked with the newly described DC3 (CD163^+^CD14^+/−^ cDC2), as it has been already shown that they can express CD206.^32^ We confirmed this using flow cytometry, which showed that CD163^+^ cDC2 (DC3) express higher levels of CD206 than CD163^−^ cDC2 (Figure S3C,D). Differential marker expression analysis showed that circulating myeloid cells in patients with PDAC and those with benign diseases presented similar phenotypic alterations compared to healthy controls, characterized by an increased expression of CD86 by classical monocytes, of MGL by cDC2 and CD272 (BTLA) by pDCs and cDC1 (Table S4). This suggests the presence of a proinflammatory component in the immune responses developed in patients with PDAC and the ones with benign disease.

Our results also revealed that PBMCs obtained from patients with PDAC and benign disease presented similar changes when compared to healthy controls, characterized by the increase of different monocyte populations (Figure 2D). Moreover, a sharp increase in the expression of CD86 in classical monocytes and CD123 in nonclassical monocytes was observed in PDAC and benign disease, supporting the idea that immune responses in PDAC present a proinflammatory component similar to benign diseases (Figure 2E,F). To identify changes associated specifically with PDAC, we repeated the analysis using the pool of healthy donors and those with benign disease (Figure 2G). This allowed us to identify that in PDAC patients, CD206^+^ cDC2 represents a higher proportion of total cDC2, which was also confirmed by flow cytometry (Figures 2G,H and S3E).

### 3.4 Pancreatic ductal adenocarcinoma is characterized by Th2‐like T cell responses

**FIGURE 3:**
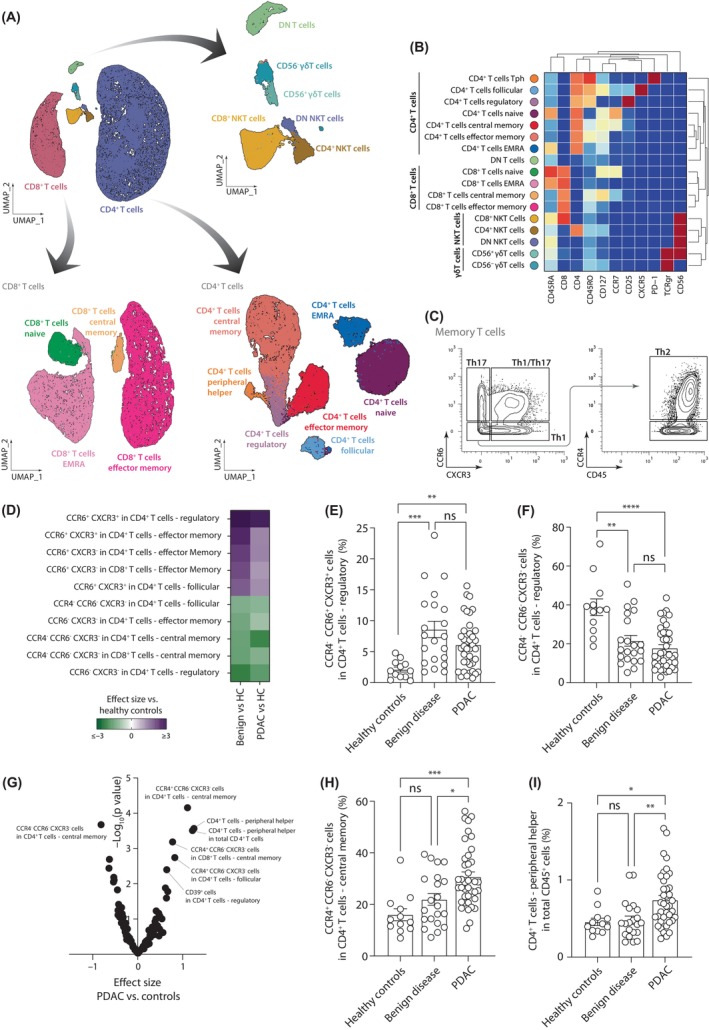
Characterization of circulating T and natural killer (NK)T cells. (A) UMAP plots with the different circulating CD3^+^ CD19^−^ cells identified. (B) Heatmap showing the expression of the different “type” markers in all the CD3^+^ subpopulations. (C) Gating strategy for the classification of memory T cells based on the expression of the chemokine receptors CCR4, CCR6, and CXCR3. (D) Heatmap summarizing the effect size of significantly abundant populations in benign disease and pancreatic ductal adenocarcinoma (PDAC) compared to healthy controls. Percentages of (E) CCR4^−^CCR6^+^CXCR3^+^ and (F) CCR4^−^CCR6^−^CXCR3^−^ in CD4^+^ regulatory T cells. (G) Volcano plot of the *p* value and the effect size of the differential cell abundance in PDAC compared to controls (healthy and benign disease). (H) Percentage of CCR4^+^CCR6^−^CXCR3^−^ in central memory CD4^+^ T cells. (I) Percentage of peripheral CD4^+^ T cells in total CD45^+^ cells. Pairwise comparisons: *p ≤ 0.05, **p ≤ 0.01, ***p ≤ 0.001, ****p ≤ 0.0001, Kruskal–Wallis test. DN, double negative; EMRA, effector memory expressing CD45RA; ns, not significant.

Subsequently, we characterized the T and NKT‐like cells present in PBMCs by analyzing CD3^+^CD19^−^ cells using our clustering pipeline and the lymphoid panel. This led to the identification of 17 immune populations, including the classical naïve and memory subpopulations in CD4^+^ and CD8^+^ T lymphocytes (Figures 3A,B and S4). We could also identify specialized populations of CD4^+^ T cells, as regulatory, follicular, and peripheral Th cells (Figures 3A,B and S4). Moreover, NKT‐like cells could be subdivided based on the expression of CD4 and CD8, while different subpopulations of γδ T cells were identified depending on expression of CD56 (Figures 3A,B and S4). The differential marker expression analysis revealed that PBMCs from patients with PDAC and those with benign disease presented similar changes when compared to the ones from healthy controls, characterized by a higher expression of FoxP3 in most of T and NKT‐like populations and a particular increase of NKG2D in CD8 T cells (Table S4). These results suggest that both groups of patients present a general activation of circulating CD3^+^CD19^−^ cells.

We also observed a great heterogeneity in the expression of the chemokine receptors CCR4, CCR6, and CXCR3 by memory T cells. Interestingly, the expression of these receptors has been associated with the polarization of the Th cells, with CCR6^+^CXCR3^−^ cells associated with Th17‐type of immune responses, CXCR3^+^CCR6^−^ cells with Th1‐type, and CCR4^+^CCR6^−^CXCR3^−^ with Th2‐type. Therefore, we also studied these different subpopulations of memory T cells according to the gating strategy shown in Figure 3C. In both groups of patients, we observed a decrease of chemokine receptor negative cells (CCR4^−^CCR6^−^CXCR3^−^), with a consequential increase of cells single and double positive for CCR6 and CXCR3, suggesting a change in the phenotype of memory T cells to Th1‐like and Th17‐like responses (Figure 3D). For example, regulatory T cells had a higher proportion of CCR6^+^CXCR3^+^ cells but a lower proportion of CCR4^−^CCR6^−^CXCR3^−^ cells in benign disease and PDAC when compared with healthy controls (Figure 3E,F). Interestingly, this phenotype switch seemed higher in benign disease than in PDAC patients (observed by a higher effect size), although no statistical differences were observed in our data (Figure 3D).

The analysis of populations specifically associated with patients with PDAC led to the identification of several CCR4^+^CCR6 CXCR3^−^ memory T cells, suggesting an increase of Th2‐like responses (Figure 3G,H). Pancreatic ductal adenocarcinoma is also associated with an increase in peripheral Th cells, characterized as CXCR5^−^PD1^hi^ CD4^+^ T cells, which have been associated with the activation of B cells in inflamed tissues (Figure 3I). To study whether this difference in phenotype also correlates with the production of cytokines by T cells, we measured the cytokine production by PBMCs after overnight stimulation with beads containing activating Abs against CD3 and CD28 (Figure S4C). In line with our results, we found that that PBMCs obtained from patients with PDAC produce a higher level of IL‐4 after stimulation, while no differences were found for IL‐17A.

Moreover, we also observed that CD39^+^ regulatory T cells were associated with PDAC (Figure 3G), driven by the difference with healthy controls (Figure S4D). These cells have a phenotype associated with effector regulatory T cells, with presence of CD45RO but lack of CD45RA, and a higher expression of the immune checkpoints TIGIT and CTLA4 than CD39^−^ regulatory T cells (Figure S4E,F). It has been previously reported that the expression of CD39 by regulatory T cells is associated with a more stable phenotype and a stronger capacity to modulate Th1 and Th17 responses.^34^, ^35^ Together, these data imply that the proinflammatory mechanisms developed in PDAC are accompanied and counteracted by the development of Th2‐like and regulatory immune responses, which may lead to less effective antitumor mechanisms.

We next studied whether these differences in the phenotype of circulating immune cells have an impact on the cytokine levels present in the serum of patients (Figure S5). Our results showed that patients with PDAC had a clear increase in the levels of IP10 (CXCL10) and MCP1 (CCL2) compared to healthy controls and patients with benign disease. A decrease in the levels of IL‐17A was observed for patients with PDAC and benign disease, although no differences were found for other T cell‐associated cytokines, such as IL‐4 and IFNγ.

### 3.5 Analysis of B, NK, and lineage negative cells

**FIGURE 4:**
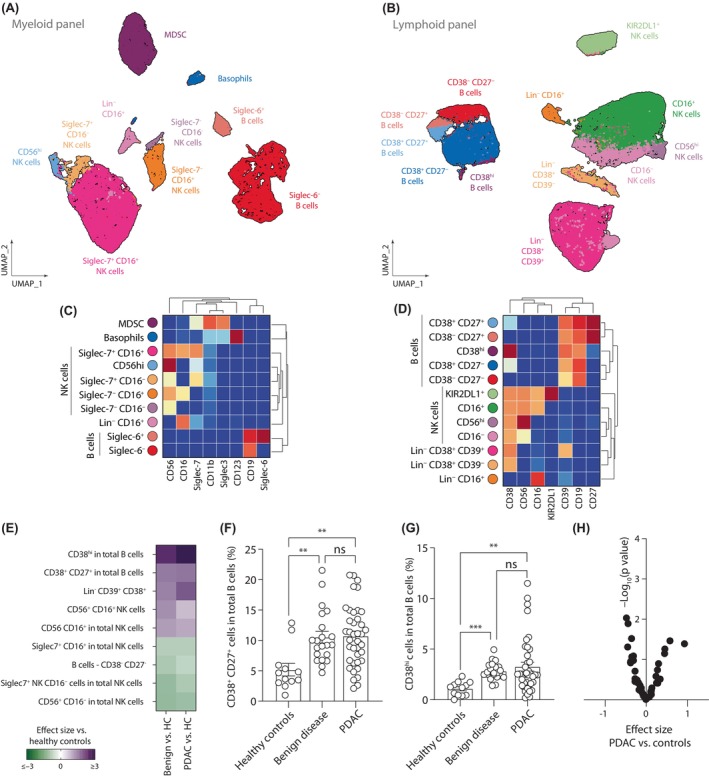
Characterization of circulating B, natural killer (NK), and lineage negative cells. (A, B) UMAP plots and (C, D) heatmaps showing the different circulating B, NK, and lineage negative cells identified using the (A, C) myeloid or (B, D) lymphoid panel. (E) Heatmap summarizing the effect size of significantly abundant populations in benign disease and pancreatic ductal adenocarcinoma (PDAC) compared to healthy controls. Percentages of (F) CD38^+^CD27^+^ cells and (G) CD38^hi^ in total B cells. (H) Volcano plot of the *p* value and the effect size of the differential cell abundance in PDAC compared to controls (healthy and benign disease). Pairwise comparisons: **p ≤ 0.01, ***p ≤ 0.001, Kruskal–Wallis test. MDSC, myeloid‐derived suppressor cell; NS, not significant.

Unlike our analysis of myeloid and T cells, which were analyzed in either the myeloid or lymphoid panel analysis, respectively, B (CD3^−^CD19^+^), NK (CD3^−^CD19^−^CD56^+^HLA‐DR^−^), and lineage negative cells (CD3^−^CD19^−^CD56^−^HLA‐DR^−^) express markers that are present in both panels (Figure S2A,B). For this reason, we performed a clustering of the gated populations together using both panels independently, which led to distinct classification of the immune cells according to the markers analyzed. For example, only two subpopulation of B cells were identified with the myeloid panel according to the expression of Siglec‐6; however, the analysis with the lymphoid panel resulted in five populations with different levels of expression of CD38 and CD27 (Figures 4A–D and S6). The latter are associated with different activation status of B cells, with CD27^−^ cells representing subtypes of naive B cells, CD27^+^ cells of memory B cells, and CD38^hi^ cells representing plasmablast‐like cells. On the other hand, both panels could identify the classical populations of NK cells based on the expression of CD16 and CD56 (Figures 4A–D and S6). However, different subpopulations of CD56^+/dim^CD16^+/−^ could be found depending on the panel used for the analysis. While the myeloid panel led to the classification of subtypes based on the expression of Siglec‐7, in the lymphoid panel a subpopulation of KIR2DL1‐expressing cells could be detected in CD56^+/dim^CD16^+^ NK cells (Figures 4A–D and S6). Finally, three populations of lineage negative cells (Lin^−^: CD3^−^CD19^−^CD56^−^HLA‐DR^−^), including Lin^−^CD16^+^ cells, were identified in both panels (Figures 4A–D and S6). Myeloid‐derived suppressor cells can be identified using the myeloid panel as Lin^−^CD11b^+^CD33/Siglec‐3^+^ (Figure 4A,C). These cells are strongly correlated with Lin^−^CD38^+^ CD37^+^ cells found in the analysis using the lymphoid panel, suggesting these cells might represent the same immune population (Figure S4).

The analysis of cell frequencies revealed several immune populations that were increased in benign disease and PDAC when compared with healthy controls, including an increase of activated subpopulations of B cells, containing CD27^+^CD38^+^ B cells and CD38^hi^ plasmablast‐like cells (Figure 4E–G). There results further support our observations that in both conditions the immune system is in a proinflammatory state. However, no PDAC‐specific populations were identified in this analysis (Figure 4H).

### 3.6 Changes in immune populations with cancer progression

**FIGURE 5:**
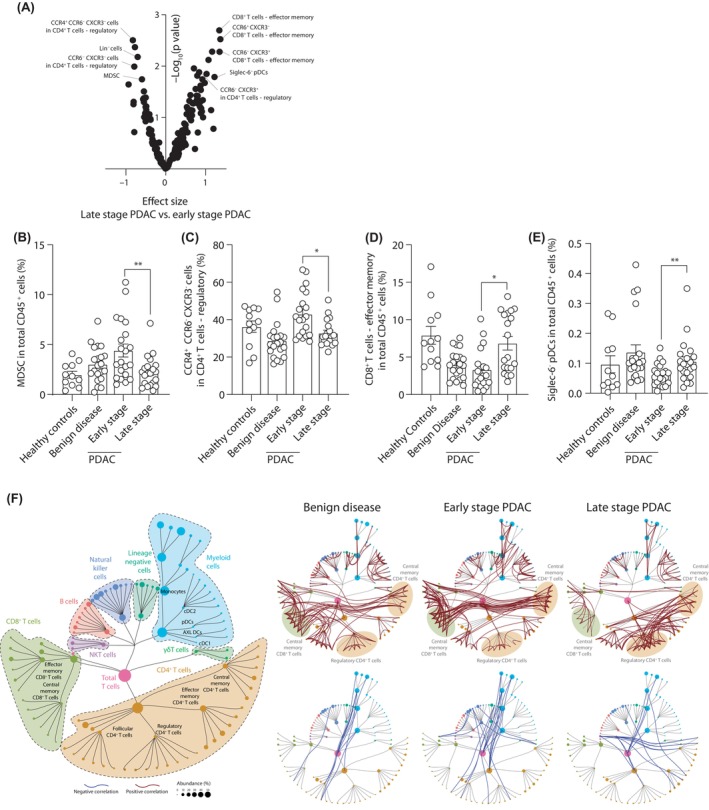
Association of immune populations with resectable and unresectable pancreatic ductal adenocarcinoma (PDAC). (A) Volcano plot of the *p* value and the effect size of the differential cell abundance in resectable and unresectable PDAC. Percentages of myeloid‐derived suppressor cells (MDSC) in (B) total CD45^+^ cells, (C) CCR4^+^CCR6^−^CXCR3^−^ cells in regulatory T cells, (D) effector memory CD8^+^ T cells in total CD45^+^ cells, and (E) Siglec‐6^−^ cells in plasmacytoid dendritic cells (pDCs). (F) Network representation of Spearman's correlation between the different populations in benign, resectable, and unresectable PDAC. Pairwise comparisons: *p ≤ 0.05, **p ≤ 0.01, Mann Whitney U‐test.

Once we characterized the immune populations present in the PBMCs from patients and identified the ones associated specifically with PDAC, we wondered about the dynamics of different populations during the progression of disease. For this, we analyzed the difference in immune populations between patients with resectable PDAC (*n* = 19) and irresectable PDAC (*n* = 19) (Figure 5, Table S5). Surprisingly, we observed that resectable PDAC correlated with the presence of immune populations associated with regulatory function, as MDSC and CCR4^+^CCR6^−^CXCR3^−^ regulatory T cells (Figure 5A–C). Controversially, late‐stage patients presented a higher presence of effector memory CD8^+^ T cells and pDCs (Figure 5D,E). These counterintuitive results may reflect the dynamics of immune cell populations migrating between blood and the cancer tissue.

The immune landscape of different pathologies is not only characterized by the presence of specific immune populations, but also by the associations between these populations. To study how the circulating immune populations are associated among themselves in the different stages of PDAC, we undertook a correlation analysis. For this, the pairwise Spearman coefficients were calculated based on the frequency of the identified immune cells on total CD45^+^ cells and significant results were plotted as a network (Figure 5F). Interestingly, we observed that patients with early stage PDAC and benign disease presented similar patterns of correlation, mainly characterized by a positive association between central memory CD4^+^ and CD8^+^ T cells (Figure 5F). This may reflect the development of proinflammatory immune responses that present both a CD4^+^ and a CD8^+^ T cell component. However, this relationship is absent in later stages of PDAC and, instead, central memory CD4^+^ T cells were strongly correlated with regulatory T cells (Figure 5F). This suggests that in latter stages of PDAC, the development of immune responses is associated with a regulatory component.

## 4 DISCUSSION

This paper shows that patients with PDAC present a multifaceted immune response: while an elevated proinflammatory component is observed in similar fashion to patients with benign disease, circulating immune cells associated to a more regulatory and protumoral immunity can also be found specifically in PDAC patients.

In myeloid cells, this is observed by a significant increase in CD86^+^ classical monocytes and CD123^+^ nonclassical monocytes in patients with PDAC and benign disease. We also showed that CD206^+^ cDC2s, also previously defined as DC3, were specifically increased in patients with PDAC.^31^, ^36^ Dutertre et al.^36^ showed that these cells exhibit a proinflammatory profile and functional activation features, including a higher capacity to prime naïve CD4^+^ T cells toward Th2 and Th17 cells. In addition, they are able to respond to Toll like receptor stimulation and secrete proinflammatory mediators, such as IL‐1β and TNFα.^36^, ^37^ Furthermore, DC3s are also able to induce the expression of CD103 in naïve CD8^+^ T cells in a transforming growth factor β‐dependent manner, and their infiltration in human breast tumor correlates with the abundance of CD103^+^ tissue T_RM_ cells.^37^ Increased frequencies of T_RM_ cells are associated with better survival in many solid cancers, however for PDAC this has not yet been examined to our knowledge.^38^, ^39^, ^40^, ^41^ Future studies could help to further unravel the role of DC3s and resident T cells in PDAC development and this might even result in novel immunotherapies.

For lymphoid cells, we found an increased expression of the cytokine receptors CCR6 and CXCR3 in memory T cells from both benign disease and tumor patients. The chemokine receptors CXCR3, CCR4, and CCR6 can be used to discriminate between different subsets of memory T cells, being associated to Th1, Th2, and Th17‐like phenotypes, respectively.^42^, ^43^ These results suggest that both patient groups develop more proinflammatory, and therefore, more antitumoral responses, characterized by the presence of circulating Th1 and Th17 T cells. Serum levels of the Th1‐associated cytokines IFNγ and TNFα have been previously associated with a better survival of PDAC patients.^44^ However, PDAC but not benign disease patients also showed an increase of CCR4^+^CCR6^−^CXCR3^−^ T cells, indicating the presence of Th2‐like immune responses. An increase in serum levels of Th2‐associated cytokines has been previously reported in patients with PDAC.^44^, ^45^ When compared to patients with chronic pancreatitis, IL‐5 was significantly increased in serum of patients with PDAC.^45^ Higher levels of circulating Th2‐associated cytokines and intratumoral Th2 cells have been shown to be associated with poor survival.^44^, ^46^ Moreover, the current study shows that patients with PDAC also present a higher expression of CD39 in regulatory T cells than healthy controls and patients with benign disease. CD39^+^ regulatory T cells have been reported to accumulate in colon cancer and are associated with stronger capacity to dampen proinflammatory responses Th1 and Th17, suggesting these cells may be able to contribute to dampen antitumor responses that arise during PDAC development.^34^, ^35^, ^47^ The coexistence of different T cell responses in PDAC patients could reflect the complex cancer biology and the acquisition of different strategies that contribute to the tumor‐induced immune escape. The presence of a proinflammatory component in the PDAC immune response is promising for the development of immunotherapies that potentiate specific Th1 responses.

This is also the first study that shows an increase in PD1^hi^CXCR5^−^ Tph cells in blood of patients with PDAC compared to those with benign disease and healthy controls. These cells have mostly been described as pathological due to their association with increased inflammation in autoimmune diseases such as rheumatoid arthritis, systemic lupus erythematosus, type 1 diabetes mellitus, and inflammatory bowel diseases.^48^, ^49^, ^50^, ^51^, ^52^, ^53^ However, the tissue counterpart of these cells was recently described by Gu‐Trantien et al. in tumors of patients with breast cancer and expansion of these cells was associated with increased overall survival and an increase in tumor‐infiltrating B cells.^54^, ^55^ Even though the total level of circulating B cells was not increased in patients with PDAC, there was a relative increase in CD38^hi^ plasmablast‐like cells. Minici et al. showed that these cells are able to sustain stroma activation by inducing collagen production by human fibroblasts, and thus contribute to the dense stroma surrounding the PDAC cells.^56^ In addition, B cells are able to attract other inflammatory cells that also have fibrotic potential such as M2 macrophages and Th2 cells.^56^ However, more studies need to be performed to determine how the Tph–B cell axis contributes to the PDAC immune escape and the clinical outcome.

Our results also showed an increase in the serum levels of the cytokines IP10 (CXCL10) and MCP1 (CCL2). Despite being considered as a proinflammatory cytokine, the role of CXCL10 in cancer progression is still unclear due to conflicting results. On one side, the expression in biopsies from pancreatic cancer patients is associated with a poor survival.^57^, ^58^ However, it was recently reported that blockade of CXCR3 (the receptor of CXCL10) in animal models of pancreatic precancerous lesions leads to more aggressive tumors.^59^ It is plausible that the induction of CXCL10 during early malignant transformation supports initial antitumor responses; but its expression in later stages, when a tolerogenic microenvironment is established, might contribute to disease progression.

In the present study, the analysis of the differential frequencies of immune cells between resectable and irresectable PDAC revealed a higher presence of memory CD8^+^ T cells in later stages of the disease. These counterintuitive results show the importance to study the infiltration of immune cells to the tumor and the correlation with peripheral blood populations. A correlation analysis between the identified immune cell populations revealed that patients with early stages of PDAC present a similar profile of connections as patients with benign disease, characterized by a positive correlation between memory CD8^+^ and CD4^+^ T cells, which may reflect the development of immune responses. However, patients with late stage PDAC presented a different network of correlations, mainly showing positive correlations between memory CD4^+^ T cells and regulatory T cells, which could indicate that the development of immune responses in later stages of the disease are associated with a regulatory component. This is important for the development of immunotherapies for PDAC, as they should not only focus to induce a potent tumor‐specific immune response, but also to overcome the regulatory responses induced by the tumor. The combination of the GVAX vaccine with the CTLA4 blocking Ab ipilimumab have shown promising results in patients.^60^ Currently, several clinical trials are being undertaken to study the combination of different vaccines with immune checkpoint blockade.^20^

Using CyTOF instead of conventional flow cytometry, we were able to analyze two extensive panels with 38 markers each, without risk of bleed‐through of the signal. Furthermore, despite being limited by the small sample size and lack of independent validation, our data serve as the first proof‐of‐concept to evaluate the potential diagnostic value of circulating immune cells in PDAC. One of the limitations of the present study is the relatively small cohort analyzed. The results should be confirmed in larger cohorts to properly assess their clinical significance. This limited the possibility to study the association of immune cells with different clinical variables important in PDAC and possible confounders, such as location of the tumor, angioinvasion, or cholestasis. As expected, there were statistically significant differences in the baseline characteristics, such as BMI, between patients with benign diseases and patients with PDAC, appropriate to the presence of PDAC. In larger cohorts, the confounding effect of these differences should be examined. However, the results presented in this report can assist in the design of flow or mass cytometry panels for immune phenotyping of PBMCs at a larger scale. In addition, not all patients underwent or required biliary drainage before blood withdrawal, influencing bilirubin levels. Furthermore, even though this study included a control group for other diseases of the pancreas and surrounding structures, the addition of samples from other malignancies such as distal cholangiocarcinoma can help to determine if the observed differences in circulating immune populations are specific for PDAC or can be extended to other cancer types.

In conclusion, patients with PDAC develop complex immune responses, characterized by the concomitant increase of cell populations associated with both anti‐ and protumoral properties. To understand better the immunological landscape in PDAC and its clinical impact, future studies should address the association between the immune cells present in peripheral blood with the local immune responses developed locally in the tumor microenvironment.

## AUTHOR CONTRIBUTIONS

**Ernesto Rodriguez:** Conceptualization; data curation; formal analysis; funding acquisition; investigation; methodology; resources; software; visualization; writing – original draft; writing – review and editing. **Eline S. Zwart:** Conceptualization; data curation; formal analysis; funding acquisition; investigation; methodology; project administration; visualization; writing – original draft; writing – review and editing. **Alsya A. Affandi:** Data curation; formal analysis; funding acquisition; methodology; software; visualization; writing – review and editing. **Jan Verhoeff:** Formal analysis; methodology; software; writing – review and editing. **Mike de Kok:** Formal analysis; methodology; software; writing – review and editing. **Lenka N.C. Boyd:** Data curation; funding acquisition; project administration; writing – review and editing. **Laura L. Meijer:** Conceptualization; data curation; funding acquisition; project administration; supervision; writing – review and editing. **Tessa Y.S. Le Large:** Conceptualization; funding acquisition; writing – review and editing. **Katarzyna Olesek:** Data curation; writing – review and editing. **Elisa Giovannetti:** Writing – review and editing. **Juan J. Garcia Vallejo:** Conceptualization; investigation; methodology; software; supervision; writing – review and editing. **Reina E. Mebius:** Conceptualization; funding acquisition; methodology; supervision; writing – review and editing. **Yvette van Kooyk:** Conceptualization; funding acquisition; supervision; writing – review and editing. **Geert Kazemier:** Conceptualization; funding acquisition; supervision; writing – review and editing.

## FUNDING INFORMATION

This work was financially supported by Immunoshape (MSCA‐ITN‐2014‐ETN No 642870) to E.R., by the Spinoza prize of the Dutch Research Council (Nederlandse Organisatie voor Wetenschappelijk Onderzoek, NWO) to Y.K., by the European Research Council (ERC‐339977‐Glycotreat) to Y.K., by the Cancer Center Amsterdam Foundation (grant number 2017‐4‐09) and the Bennink Foundation (grant number 2005619) to E.Z and G.K., and the Associazione Italiana per la Ricerca sul Cancro (AIRC) Start‐Up grant to E.G.

## CONFLICT OF INTEREST STATEMENT

The authors have no conflict of interest to declare.

## ETHICS STATEMENTS

Approval of the research protocol by an institutional review board: The study design and protocol were approved by the local Medical Ethics Board of the Amsterdam UMC, VU University Amsterdam.

Informed consent: Informed consent was obtained from the subject(s) before participation.

Registry and the registration no. of the study/trial: VUMC 2012;59.

Animal studies: N/A.

## Supporting information


Figure S1

Table S1
