# Supplementary material for: In‐depth immune profiling of peripheral blood mononuclear cells in patients with pancreatic ductal adenocarcinoma reveals discriminative immune subpopulations
Source: Cancer Sci. 2024 Apr 30;115(7):2170–83. doi: 10.1111/cas.16147 (PMC11247553; doi:10.1111/cas.16147)
Supplement: Supplementary file 1 — Figure S1 [file CAS-115-2170-s001.docx]

**SUPPLEMENTARY METHODS**

**CyTOF Antibody Labeling and Titration**

Antibody labeling with the indicated metal tag was performed using the MaxPAR® antibody conjugation kit (Fluidigm), according to manufacturer instructions. In short, 100μg antibody for the lanthanide conjugated antibodies and 50 μg antibody for the cisplatin conjugated antibody were washed with R-buffer using a 50kDa Eppendorf filter (Millipore) before 100μL of 4mM TCEP-R-Buffer was added and incubated at 37°C for 30 minutes. Next, filters were washed with R-Buffer followed by a wash with C-Buffer. Simultaneously, the polymer was resuspended with 95μL L-Buffer, followed by adding 5μL of lanthanide metal solution. This mix was incubated at 37°C for 35 minutes. The lanthanide-loaded polymer was washed with L-buffer using a 3kDa Eppendorf filter followed by a wash with C-buffer. Both the lanthanide-loaded polymer and the partially reduced antibody were mixed in the 50kDa filter, incubated for 90 minutes at 37°C, and collected by inverting the 50kDa filter over to a new collection tube and centrifuging at 1000g for 2 minutes. For the cisplatin conjugated antibody, the reduced antibody was washed with C-Buffer and spun down inverted at 1000g for 2 minutes. The remaining antibody was washed from the filter with C-buffer. To this reduced antibody, 20μL cisplatin was added and incubated for 90 minutes at 37°C. Purification of the bound antibody was performed with the high-performance liquid chromatography (ThermoFisher) and subsequently concentrated by filtering with a 10kDa filter (Merck KGaA, Darmstadt Germany Millipore). The end volume was determined and an equal volume of antibody stabilizer (Candor Bioscience) was added before the antibodies were stored at 4°C. All antibodies used in this study were titrated using both fixed and unfixed thawed PBMCs. The most optimal concentration with the least spillover was chosen. To avoid pipetting errors between batches, a master antibody cocktail was made, aliquoted and stored in -80°C, as previously described.^1^

**CyTOF Staining Protocol and sample acquisition**

First, cells were counted after the thawing process. From each patient, 6x10^6^ cells were stained for 10 minutes at RT with 1:1000 Cell-ID Intercalator-^103^Rh (Fluidigm) as live dead marker, followed by Fc blockade (Biolegend) for 30 minutes at RT to prevent nonspecific binding. The cells were split into two plates for both the lymphoid and myeloid panel. Next, markers which are affected by fixation, as determined by the titration, were stained. Subsequently, cells were fixated with 1.6% fresh paraformaldehyde (PFA), barcoded and stained cells from different patients were mixed. Cell mix was counted, stained with the remainder surface antibodies, followed by re-fixating with 1.6% fresh PFA. Staining of intra-nuclear markers was performed after incubation with Nuclear Staining buffer (Fluidigm) and finally fixation with Fix and Perm buffer (Fluidigm) containing Iridium (Fluidigm) overnight at 4°C. Fixed cells were washed twice with CSB and divided in approximately 1x10^6^ cells per tube, followed by washing with MilliQ water right before acquisition. Calibration beads were added to the suspension in a total volume of 15%. Cells were acquired on the Helios™ (Fluidigm), with an event rate of approximately 250-350 events per second.

**Cytometry Data Processing**

Acquired samples were randomized using Gaussian negative half zero randomization in Cytof Software version 6.7. The FCS files were bead normalized (as described by Finck *et al*) in R using the function *normCytof* as applied in the package *CATALYST*.^2, 3^ Stable signal was selected using the R package *Flowcut* and files where then debarcoded using the package *CATALYST*.^4^ Quantile normalized per batch based on the reference samples was performed using the R package *CytoNorm*.^5^ Cleanup of the data was performed as described by Fluidigm until CD45^+^ cells^6^. The quantification of immune populations was performed by manual gating, performed in Flowjo version 10.

**Flow cytometry**

Thawed and washed PBMCs were seeded in duplicated in a 96 well plate and let them rest for three hours at 37°C. Cells were washed and incubated with Fc-block and viability dye prior to cell surface staining with fluorescence-conjugated antibodies in 0.5% BSA/PBS for 20 min at 4°C. The used antibodies can be found in Supplementary Table 2. Cells were first incubated with the surface stain antibodies. After thorough washes and permeabilization with 0.5% PFA, cells were incubated in 0.5% BSA/0,5% saponin/PBS with the intracellular antibodies. Finally, cells were fixed with 2% paraformaldehyde for 10 min at 4°C and stored.


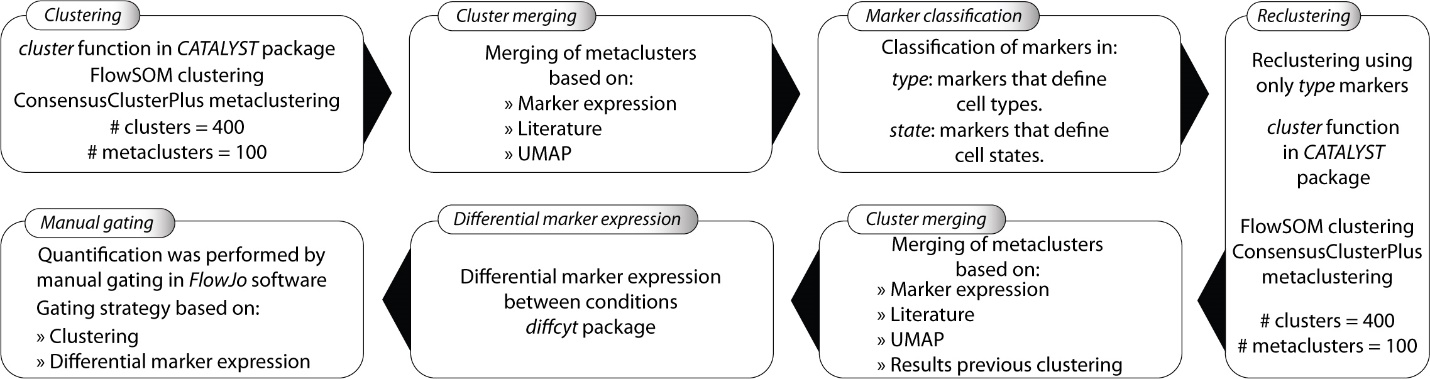


**Supplementary Figure 1. Workflow used for the identification of cell types.**


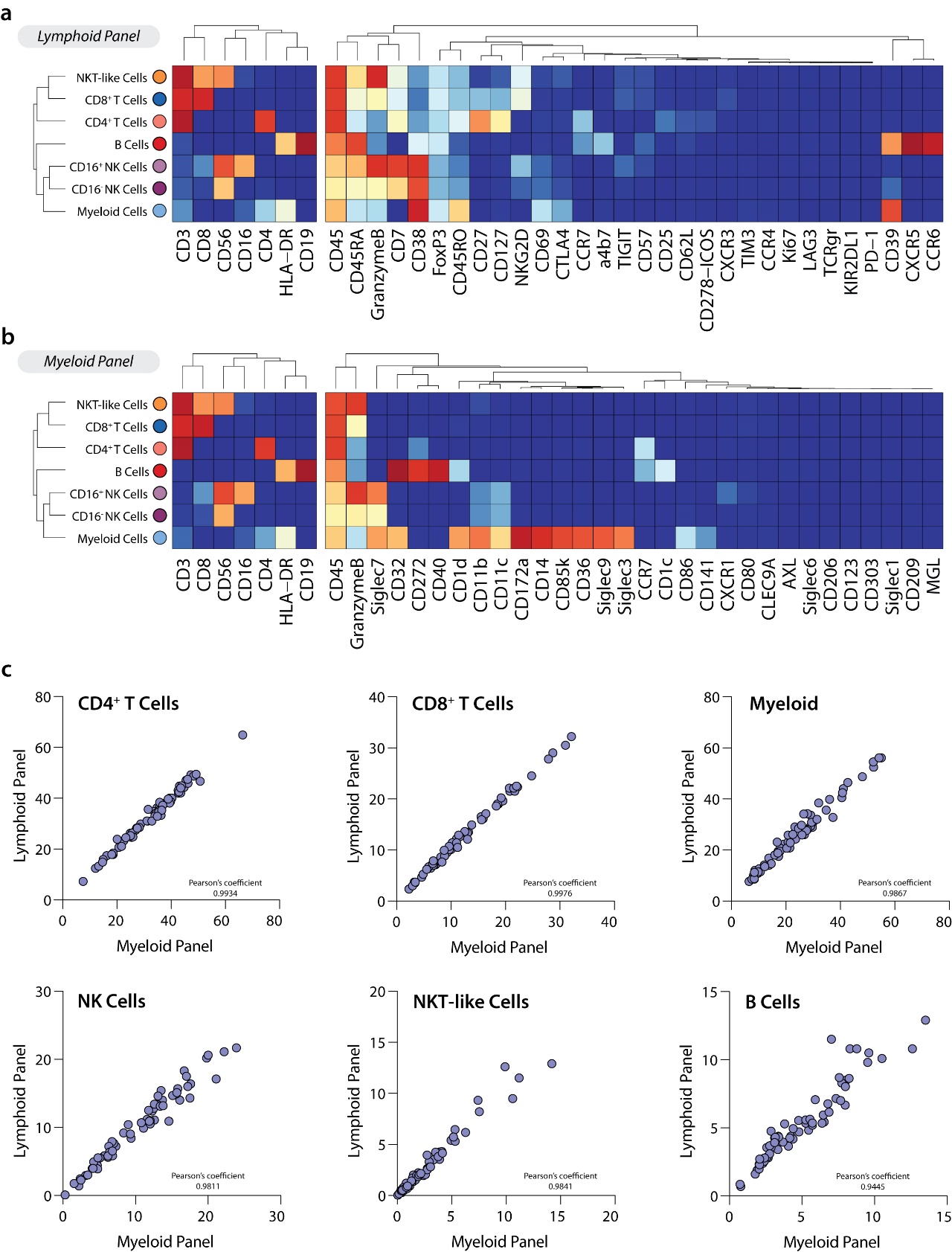


**Supplementary Figure 2. Characterization of general populations using a Lymphoid and a Myeloid panel.** Heatmaps showing the expression of common markers (left) and other markers (right) by the different general populations using the *Lymphoid* (a) or *Myeloid* (b) panel. c) Correlation between the quantification of general populations using the *Lymphoid* and *Myeloid* panel.


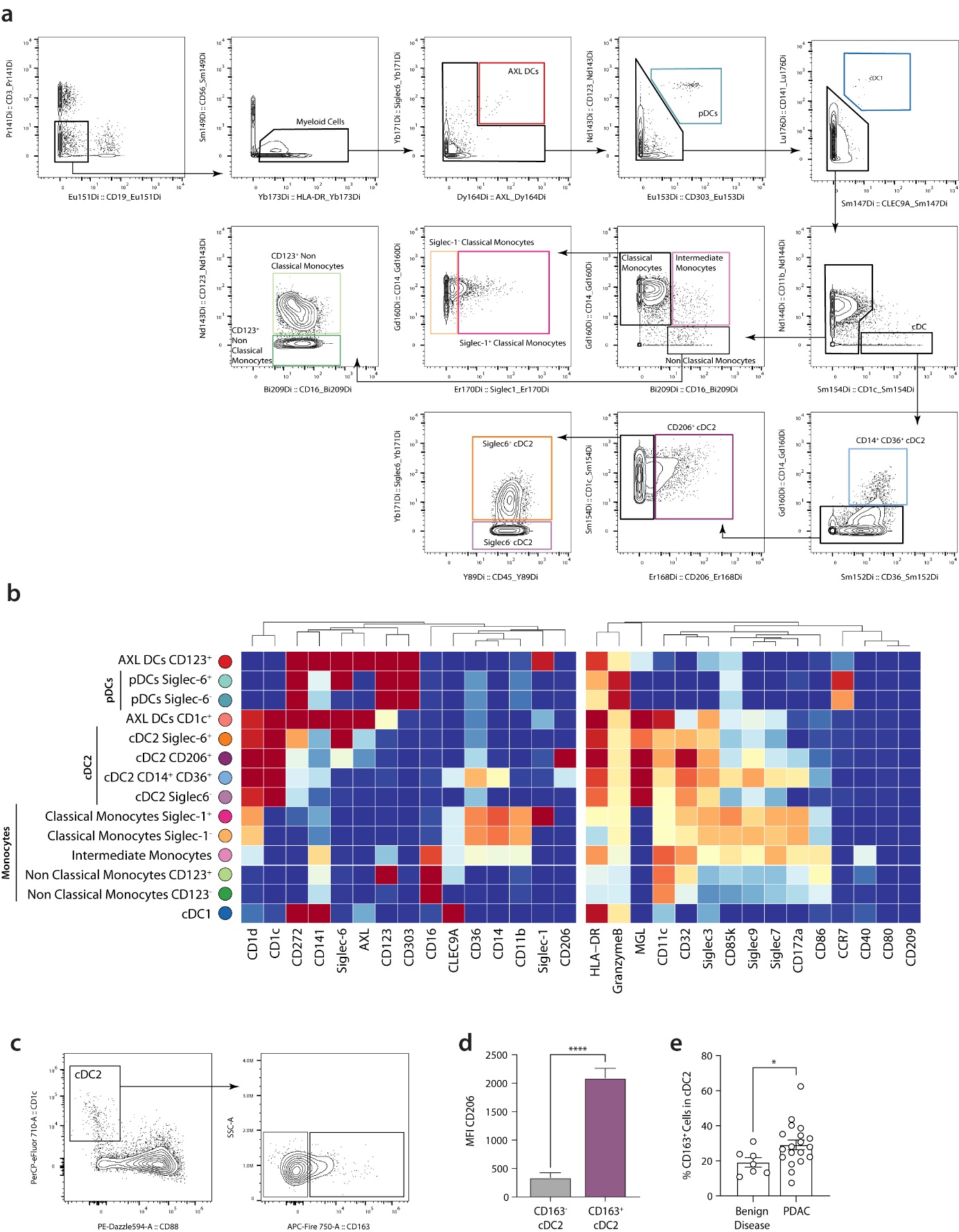


**Supplementary Figure 3.** a) Gating strategy used for the different myeloid populations. b) Heatmap displaying the expression of *type* (left) and *state* (right) markers in myeloid populations. c) Gating strategy for the analysis of CD163+ cDC2 using flow cytometry. d) Expression of CD206 (Mannose receptor) in CD163 positive and negative cells. e) Quantification of CD163+ cells in total cDC2 using flow cytometry. Kruskal-Wallis test (*p ≤ 0.05, **p ≤ 0.01, ***p ≤ 0.001, ****p ≤ 0.0001).


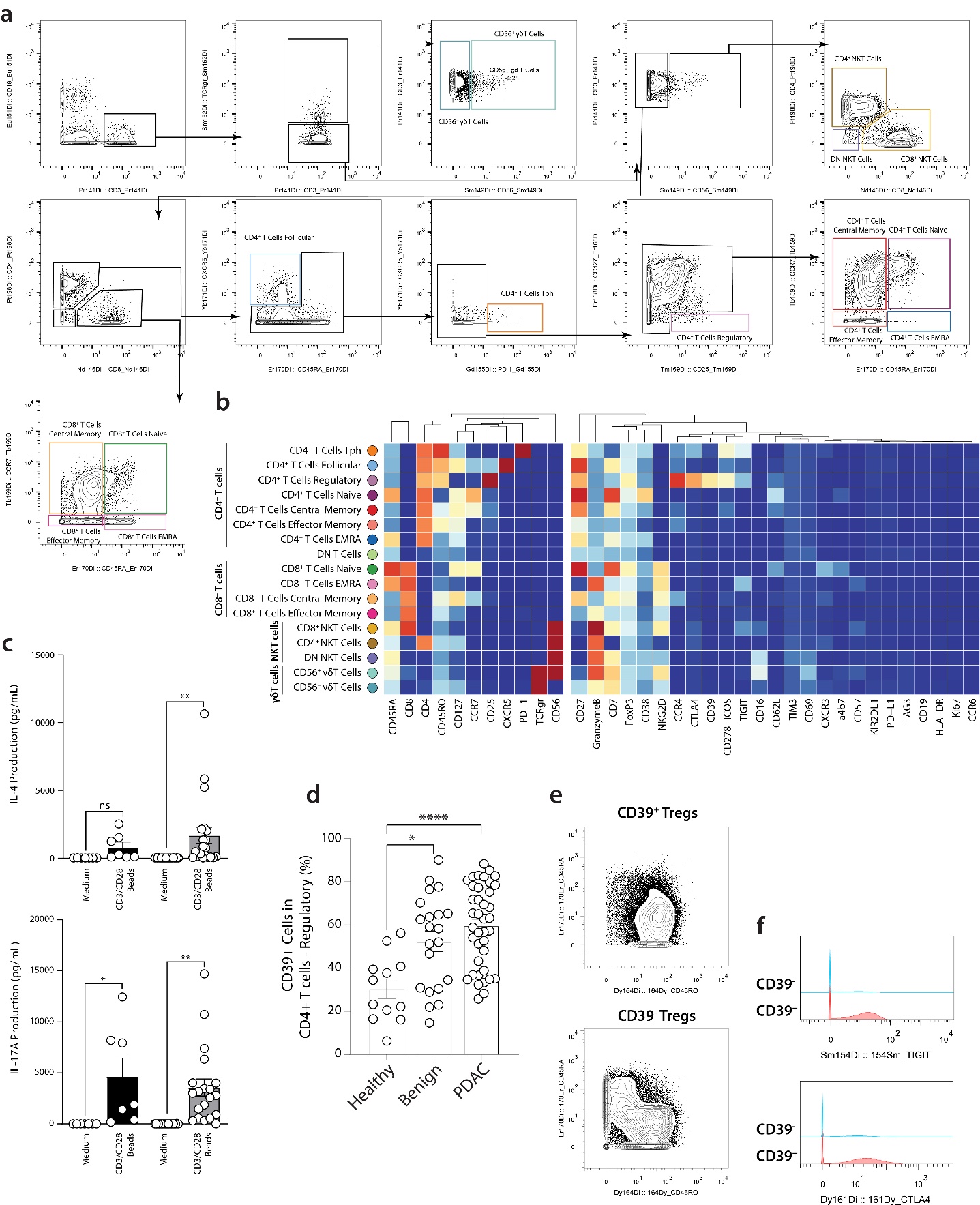


**Supplementary Figure 4.** a) Gating strategy used for the different lymphoid populations. b) Heatmap displaying the expression of *type* (left) and *state* (right) markers in lymphoid populations. c) Cytokine production by PBMCs after overnight stimulation of PBMC with CD3/CD28 beads. d) Quantification of CD39^+^ cells in regulatory CD4 T cells. Pairwise comparisons: Kruskal-Wallis test with Dunn multiple comparisons test (*p ≤ 0.05, **p ≤ 0.01, ***p ≤ 0.001, ****p ≤ 0.0001). Expression of CD45RA and CD45RO (e), TIGIT and CTLA4 (f) in CD39^+^ and CD39^-^ regulatory T cells.


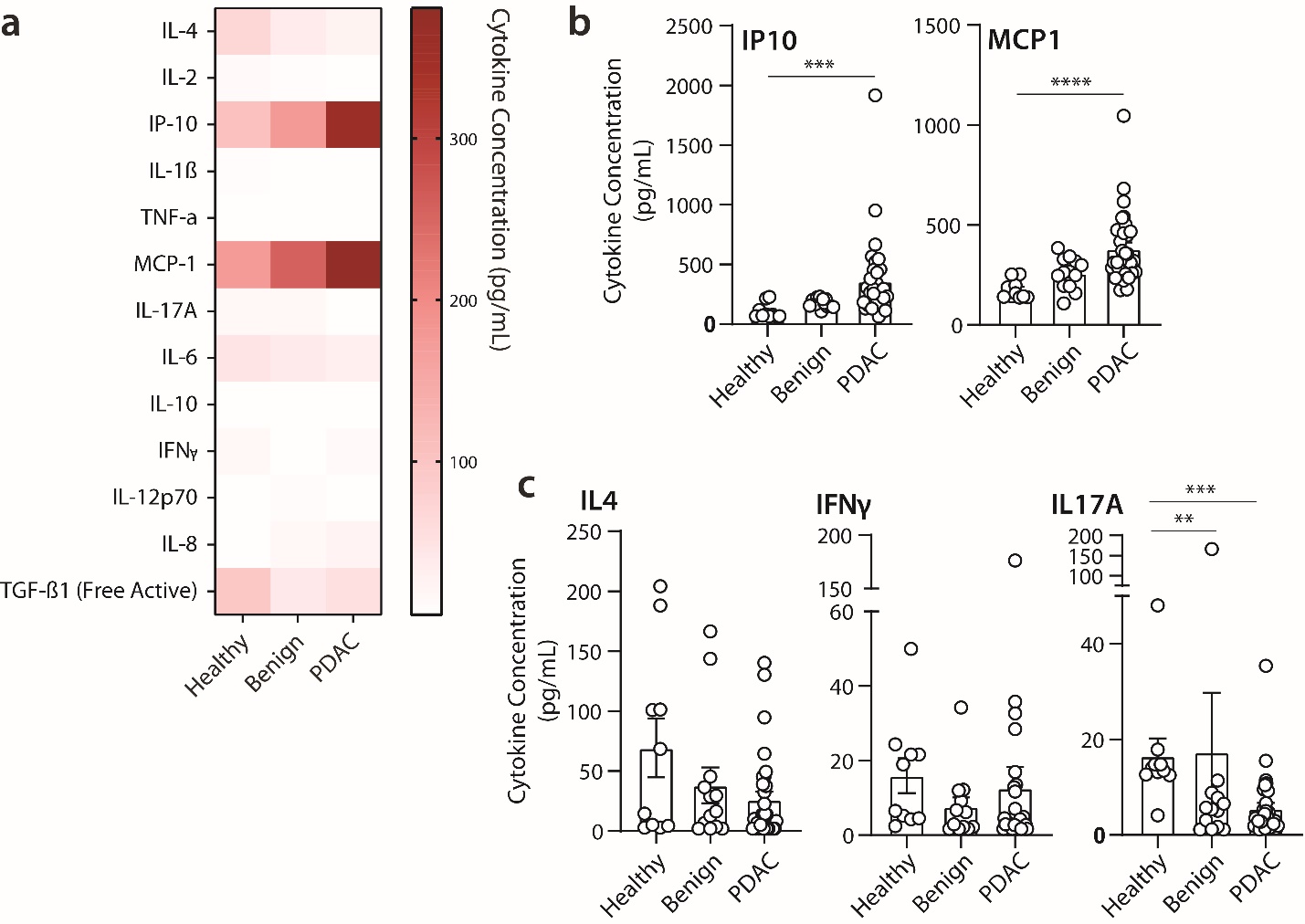
**Supplementary Figure 5. Cytokine levels in serum.** a) Heatmap showing the concentration of cytokines in the serum of patients. b) Concentration of IP10 (CXCL10) and MCP1 (CCL2). c) Concentration of cytokines associated with T cell function (IL-4, IFNγ, IL17A). Pairwise comparisons: Kruskal-Wallis test (*p ≤ 0.05, **p ≤ 0.01, ***p ≤ 0.001, ****p ≤ 0.0001)


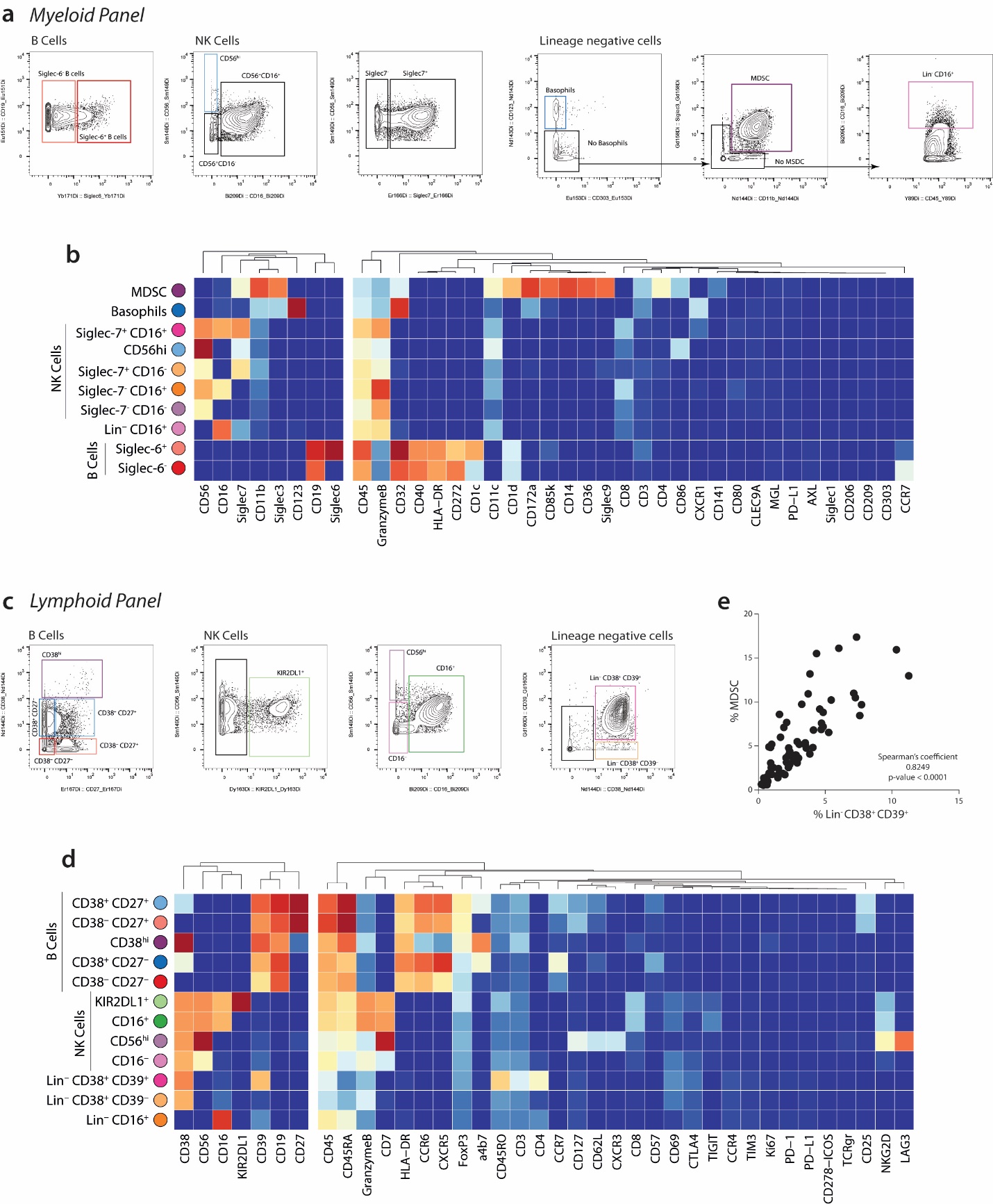


**Supplementary Figure 6.** Gating strategy used for B, NK and lineage negative cells populations using the Myeloid Panel (a) or the Lymphoid panel (c). Heatmap displaying the expression of *type* (left) and *state* (right) markers in B, NK and lineage negative cells using the *Myeloid Panel* (b) or the *Lymphoid panel* (d). e) Correlation between the frequency of MDSC identified in the *Myeloid Panel* and the Lin^-^ CD38^+^ CD39^+^ cells in the *Lymphoid Panel*.

**SUPPLEMENTARY REFERENCES**

1. Schulz AR, Baumgart S, Schulze J, et al. Stabilizing Antibody Cocktails for Mass Cytometry. Cytometry A 2019;95:910-916.

2. Finck R, Simonds EF, Jager A, et al. Normalization of mass cytometry data with bead standards. Cytometry A 2013;83:483-94.

3. Crowell H, Zanotelli V, Chevrier S, et al. CATALYST: Cytometry dATa anALYSis Tools. R package version 1.18.1, 2022.

4. Meskas J, Wang S, Brinkman R. flowCut — An R package for precise and accurate automated removal of outlier events and flagging of files based on time versus fluorescence analysis. bioRxiv 2020:2020.04.23.058545.

5. Van Gassen S, Gaudilliere B, Angst MS, et al. CytoNorm: A Normalization Algorithm for Cytometry Data. Cytometry A 2020;97:268-278.

6. Bagwell CB, Inokuma M, Hunsberger B, et al. Automated Data Cleanup for Mass Cytometry. Cytometry A 2020;97:184-198.
